# Equipartitioning of Molecular Degrees of Freedom in MD Simulations of Gaseous Systems via an Advanced Thermostatization Strategy

**DOI:** 10.1021/acs.jctc.4c01580

**Published:** 2024-12-19

**Authors:** Jakob Gamper, Josef M. Gallmetzer, Risnita Vicky Listyarini, Alexander K. H. Weiss, Thomas S. Hofer

**Affiliations:** † Theoretical Chemistry Division, Institute of General, Inorganic and Theoretical Chemistry, Center for Chemistry and Biomedicine, University of Innsbruck, Innrain 80-82, A-6020 Innsbruck, Austria; ‡ Research Institute for Biomedical Aging Research, University of Innsbruck, Rennweg 10, A-6020 Innsbruck, Austria

## Abstract

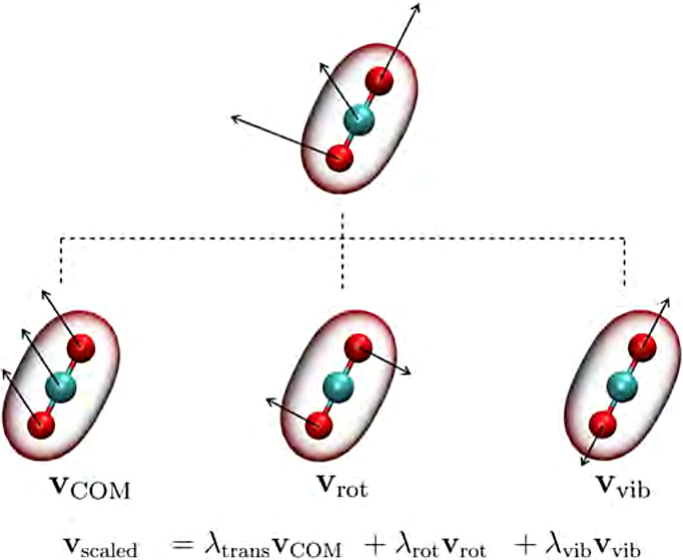

This work introduces a dedicated thermostatization strategy for molecular dynamics simulations of gaseous systems. The proposed thermostat is based on the stochastic canonical velocity rescaling approach by Bussi and co-workers and is capable of ensuring an equal distribution of the kinetic energy among the translational, rotational, and vibrational degrees of freedom. The outlined framework ensures the correct treatment of the kinetic energy in gaseous systems, which is typically not the case in standard approaches due to the limited number of collisions between particles associated with a large free mean path. Additionally, an efficient strategy to effectively correct for intramolecular contributions to the virial in quantum mechanical simulations is presented. The equipartitioning thermostat was successfully tested by the determination of pV diagrams for carbon dioxide and methane at the density functional tight binding level of theory. The results unequivocally demonstrate that the equipartitioning thermostat can effectively achieve an equal distribution of the kinetic energy among the different degrees of freedom, thereby ensuring correct pressure in gaseous systems. Furthermore, RDF calculations show the capability of the proposed method to accurately depict the structure of gaseous systems, as well as enable an adequate treatment of gas molecules under confinement, as exemplified by an MD simulation of (CO_2_)_50_@MOF-5.

## 1 Introduction

The rapidly accelerating global climate change is widely regarded as one of the greatest challenges of the new millennium, as the associated rise in global mean temperature is already having a major impact on ecosystems and human communities.^1,2^ The continued emission of greenhouse gases such as CO_2_, CH_4_ and N_2_O has been identified as one of the major factors contributing to the significant changes in global climate already observable today.^3^ As a result, research activities are increasingly focusing on carbon capture and storage/sequestration (CCS)^4,5^ on the one hand, and on the formulation of novel energy technologies^6,7^ on the other. Among others, the storage of greenhouse gases such as CO_2_ and N_2_O as well as technologically relevant energy carriers such as H_2_ and NH_3_ in suitable adsorption media such as zeolites, activated carbon as well as metal and covalent-organic frameworks (MOFs/COFs) has received increased attention in recent years.^8,9^

Due to the broad variability in composition, MOFs and COFs offer a wide range of properties for different applications.^10−12^ While numerous MOF and COF materials have been investigated for their gas storage capacity, the almost limitless possibilities in formulating new compounds make further testing a tedious, time-consuming and expensive approach.

An alternative to experimental investigations are computational studies, which are capable of screening a wide variety of compounds even before they have been synthesized in the lab. Indeed, numerous studies have been reported focusing on the investigation of gaseous guest molecules incorporated into suitable host matrices such as MOFs and COFS, using either force field (FF) based approaches,^13−16^ semiempirical methods,^17−19^ density functional theory^20−22^ as well as neural network potentials.^23,24^ These approaches have been used with great success to study a wide range of properties of these gas@host systems.

**Figure 1:**
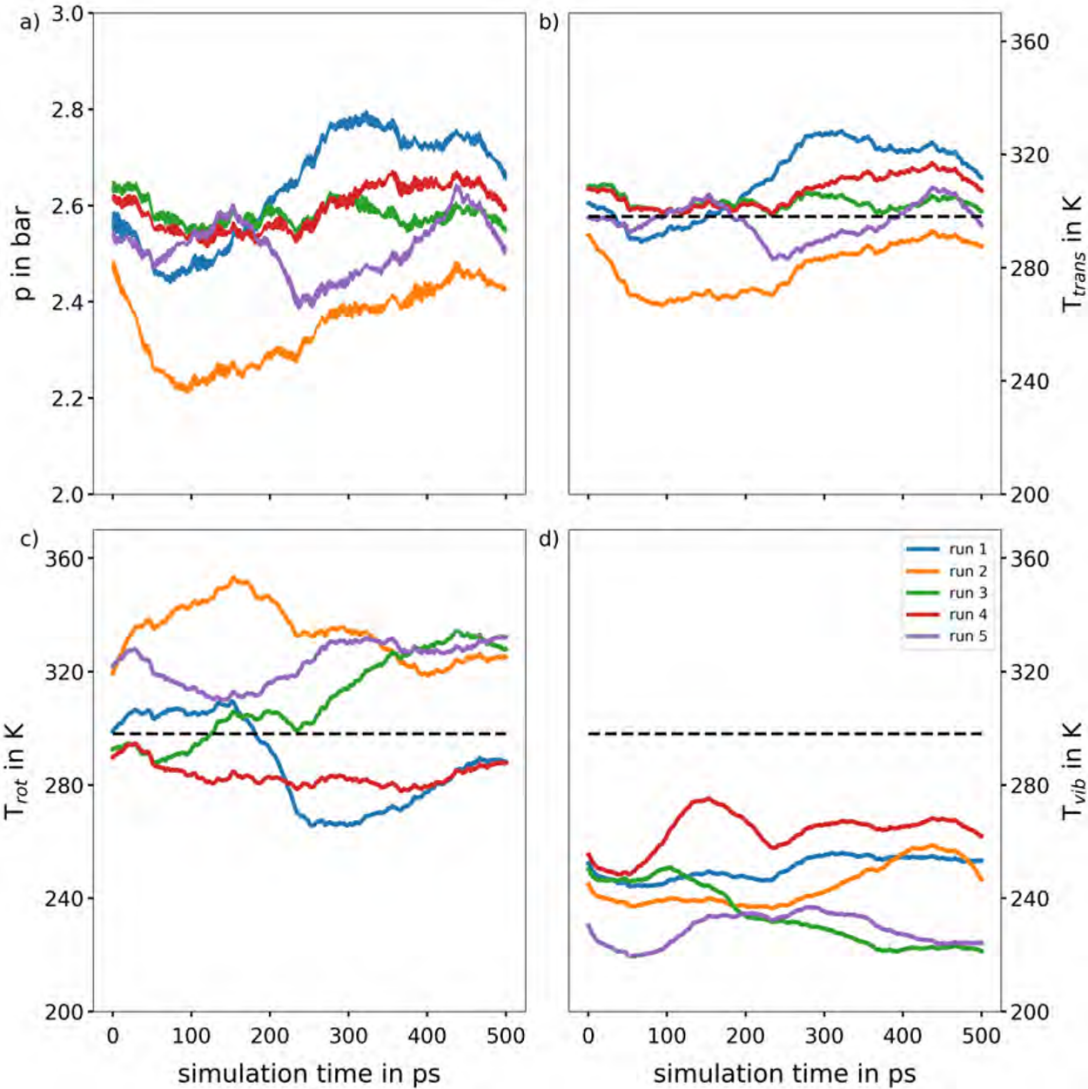
Time evolution of (a) pressure as well as (b) the translational, (c) the rotational and (d) the vibrational temperature obtained from five different NVT QM-MD simulations of gaseous CO_2_ at DFTB3/3ob/D3 level of theory applying the stochastic canonical velocity rescaling thermostat^29^ to a cubic simulation cell with a box length of 100.9597 Å. The CO_2_ molecules were placed randomly in the simulation box and the velocities were initialized according to the Maxwell–Boltzmann distribution at 298.15 K corresponding to the target temperature (black dashed line).

However, some quantities require an adequate description of a suitable reference state, which in this context corresponds to a simulation of the unperturbed gas. While the solid and liquid states are typically considered much more difficult to describe theoretically due to their higher density, the correct treatment of a gas employing a finite number of molecules is surprisingly difficult to achieve in a molecular dynamics (MD) simulation setting. This is demonstrated in the following using an MD simulation of pure carbon dioxide treating 50 CO_2_ molecules in a periodic simulation cell of fixed volume *V* at the density functional tight binding (DFTB) level of theory.^25,26^Figure 1a depicts the time evolution of the associated pressure when starting the simulation from five different randomized initial geometries. The latter have been constructed ensuring (i) Boltzmann distributed initial velocities corresponding to 298.15 K and (ii) an equipartitioned distribution of the kinetic energy according to the degrees of freedom associated with translational, rotational and vibrational motion. As can be seen each simulation results in a notably different evolution of the instantaneous pressure *p*.

In MD simulations the latter is typically determined based on the virial theorem^27^ given as
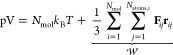
1with *N*_mol_, *N*_atoms,i_, *k*_B_ and *T* being the number of gas molecules, the number of atoms per gas molecule *j*, Boltzmann’s constant and the instantaneous system temperature *T*, respectively. The latter term given as the sum of scalar products of the force and position vectors **F**_*ij*_ and **r**_*ij*_ is commonly referred to as virial .^27^ In context of periodic QM calculations the contributions to  can be directly obtained from the diagonal elements of the associated stress tensor **σ**.^28^

At sufficiently high temperatures *T* and low densities the contribution arising from the virial is to a large extend negligible, i.e., carbon dioxide can be expected to display properties effectively corresponding to an ideal gas at ambient conditions. This implies that the differences in pressure observed in the different simulations shown in Figure 1a are due to variations in temperature. The latter is linked to the kinetic energy of the system *E*_kin_ via
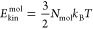
2Here, the molecular (i.e., center-of-mass) kinetic energy  has to be employed since the calculation of the pressure is carried out considering molecules as individual units.

The molecular kinetic energy  for a single molecule *i* can be obtained in a straightforward manner by summing the momenta of the individual atoms *j* according to
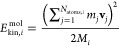
3with *m*_*j*_ and **v**_*j*_ being the mass and velocity of atom *j*, *N*_atom,*i*_ and *M*_*i*_ correspond to the number of atoms and the total mass of molecule *i*, respectively. The molecular kinetic energy is only composed of contributions arising from the translational degrees of freedom since momentum components arising due to rotational and vibrational motions cancel.

Thus, the fact that different pressures are observed in the example depicted in Figure 1a already points toward inconsistencies in the distribution of the kinetic energy among the individual degrees of freedom (DOF). This is verified when considering the contributions of the total kinetic energy obtained after decomposition according to translational, rotational and vibrational motion shown in Figure 1b–d (see Section 2 for details on the decomposition procedure). Clearly, the expected distribution of the kinetic energy according to the equipartition theorem is not achieved, resulting in different effective temperatures that are strongly dependent on the employed initial structure. Moreover, collisions between gas molecules often result in a redistribution of kinetic energy, which can amplify the problem. While due to the comparably long mean free path of gas molecules a similar situation may indeed occur in nature, the large number of molecules on the molar scale can be expected to compensate an uneven distribution of *E*_kin_ when considering the entire ensemble. However, since it is not feasible to execute simulations employing such an exceptionally high number of molecules at QM and semi-empirical level of theory, the equipartitioning of the kinetic energy has to be enforced via a suitably adjusted thermostatization algorithm.

In the following, an equipartitioning thermostat focused on simulations of gas phase systems is presented, that is based on the stochastic canonical velocity rescaling approach by Bussi and co-workers.^29^ It can be shown, that based on a separate thermostatization of translational, rotational and vibrational velocity components, the equipartitioning of the kinetic energy can be achieved without any negative impact on the simulation.

Even when an equal distribution of the kinetic energy among the different degrees of freedom is achieved, intramolecular contributions to the virial  may further complicate the evaluation of the pressure. While it is straightforward to eliminate these contributions in pairwise additive force fields, separating the potential energy in intra- and intermolecular components, intramolecular contributions may well be present in QM-based simulations^28^ as well as polarizable, reactive force field approaches such as ReaxFF.^30^

Thus, in addition to formulating an equipartitioning thermostat, an efficient strategy to correct for intramolecular contributions to the virial  had to be developed in this work.

## 2 Methods

### 2.1 The Equipartition Theorem

The equipartition theorem, a fundamental principle in classical statistical mechanics, states that within a system at thermal equilibrium, the average kinetic energy is distributed equally among all accessible degrees of freedom. In general, for a system of *N* individual atoms with *f*_tot_ = 3*N* – 3 degrees of freedom, the average kinetic energy per degree of freedom  is given by
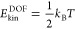
4It is crucial to differentiate between the various types of kinetic energy associated with the different degrees of freedom being translational, rotational and vibrational. The total kinetic energy of the system can be expressed as the sum of those individual contributions:
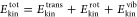
5with ,  and  being the translational, rotational and vibrational kinetic of the system, respectively.

### 2.2 Weak Coupling Thermostat

A widely used thermostatization strategy in molecular dynamics simulations, is the velocity rescaling approach, in which the velocities obtained after time propagation are adjusted using a suitable scaling factor λ according to
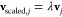
6
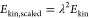
7with **v**_*j*_ and *E*_kin_ being the velocity of atom *j* and kinetic energy of the system, respectively. In general, both, the Berendsen^31^ and the stochastic canonical velocity rescaling thermostat by Bussi et al.^29^ are based on the assumption that the system is weakly coupled to an external heat bath. To obtain a velocity distribution consistent with a canonical ensemble, Bussi et al. developed an extension of the Berendsen weak coupling approach by adding a stochastic contribution based on a Wiener noise d*W* (i.e., a random number drawn from a Gaussian distribution with zero mean and unit variance) to the determination of λ, resulting in the following expression for the auxiliary dynamics
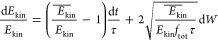
8with τ and *f*_tot_ being the coupling constant to the external heat bath, the total number of degrees of freedom and *E*_kin_® is the target kinetic energy. Furthermore, both thermostatization strategies uniformly modify the velocities of all particles in a system with the same scaling factor, thus directly influencing the overall temperature of the system.

The strategy of the presented equipartitioning thermostat is to apply independent stochastic canonical velocity rescaling thermostats to the translational, rotational and vibrational degrees of freedom in order to achieve an equal distribution of the kinetic energy among all degrees of freedom within a much shorter time scale. This is particularly necessary for the treatment of gaseous systems using quantum chemical approaches, in which the number of collisions between the particles is comparatively low, thus resulting in a slow redistribution of the kinetic energy between the different degrees of freedom.

### 2.3 The Equipartitioning Thermostat

In order to determine the translational, rotational and vibrational temperatures of a system in MD simulations, a decomposition of the velocities of all molecular entities is necessary. In MD simulation approaches a single molecular entity can be considered as a molecular unit, which especially holds true for gaseous systems, where the intermolecular interactions are comparatively weak. Thus, the translational kinetic energy can be expressed via the center-of-mass velocity of the molecule **v**_COM_ as
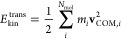
9where *m*_*i*_ is the mass of the *i*-th molecule and *N*_mol_ is the total number of molecules in the system.

The rotational kinetic energy can be expressed as
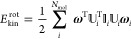
10where _*i*_ is a unitary matrix that diagonalizes the moment of inertia tensor _*i*_ to yield the eigenvalues of the principal moments of inertia and **ω**_*i*_ is the angular velocity vector of the *i*-th molecule. The superscript T denotes the respective vector/matrix transpose.

When considering the vibrations of a molecular system, only motions with zero linear and angular momentum contribute. Therefore, the vibrational kinetic energy can be represented as the remaining velocity contribution not accounted for in the translational and rotational kinetic energy components. When transforming the angular velocity vector ω to a linear velocity vector **v**_rot_ the vibrational kinetic energy takes the following form
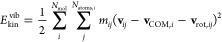
11with *N*_mol_ being the number of molecules in the system and *N*_atoms,*i*_ the number of atoms associated with the *i*-th molecule with *m*_*ij*_ and **v**_*ij*_ being the mass and velocity of the *j*-th atom in the molecule. In order to apply a thermostat based on effective translational, rotational and vibrational temperatures, the respective degrees of freedom have to be considered separately for each molecular species. Generally, one molecule features a total of 3*N*_atoms_ degrees of freedom *f*_tot_. (i) In case of a monatomic gas (e.g., Ar), the rotational and vibrational DOF are zero, thus resulting in a total of three translational DOF *f*_trans_ only. (ii) In case of a linear molecule, the rotational DOF *f*_rot_ are reduced by one, thus resulting in three translational and two rotational DOF. (iii) In case of a nonlinear molecule, a total of three translational and three rotational DOF can de derived. (iv) The vibrational DOF *f*_vib_ always represents the remaining DOF that do not contribute to the translational and rotational motion of the molecule. Including these considerations the effective translational, rotational and vibrational temperatures can be expressed as
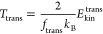
12
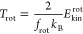
13
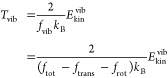
14

### 2.4 Velocity Decomposition

Regarding the implementation of the equipartitioning thermostat, the decomposition of the velocities of all molecules in its translational, rotational and vibrational components is necessary. First, for the determination of the translational kinetic energy the center-of-mass velocity *v*_COM,*i*_ has to be calculated for each molecule *i* as
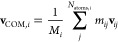
15with *M*_*i*_ being the total mass molecule *i*, *m*_*ij*_ and **v**_*ij*_ are the mass and velocity of the *j*-th atom of the molecule, respectively. Based on all **v**_COM,*i*_ in the system, the effective translational temperature can be determined. Afterward, the Bussi et al. velocity rescaling formulation^29^ is applied to determine the global scaling factor λ_trans_, which is then used to scale the translational velocities of all molecules in the system according to eq 7.

To address the scaling of the rotational temperature of the system, it is necessary to determine the rotational velocity and corresponding angular velocity of each molecule. First, the rotational velocity of each atom in a molecule can be described as
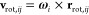
16
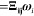
17where **v**_rot,*ij*_ is the rotational velocity and **r**_rot,*ij*_ the rotational position vector of a single atom *j* along the principal axis of inertia of molecule *i*. The vector **ω**_*i*_ corresponds to the angular velocity of molecule *i* and matrix Ξ_*ij*_ represents a unique projection operator for each atom. The formulation of the latter is adopted from Hansen and Taub^32^ and is discussed in the following.

To obtain the atomic rotational velocities, the principal axes of inertia vectors **e**_1,*i*_, **e**_2,*i*_ and **e**_3,*i*_ (i.e., the eigenvectors of _*i*_) have to be determined for each molecule *i* via diagonalization of the respective molecular _*i*_. The latter can be expressed as
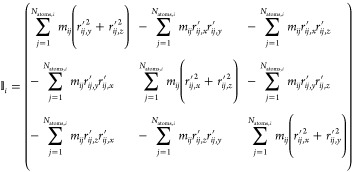
18where *r*_*ij*,*x*_*′*, *r*_*ij*,*y*_*′* and *r*_*ij*,*z*_*′* are the components of the position vector of atom *j* in the center-of-mass frame of molecule *i*. Then, to obtain the rotational velocity **v**_*ij*,rot_ of atom *j* its relative velocity to the center-of-mass velocity of molecule *i* has to be projected onto the principal axes of inertia of the molecule via
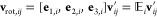
19where **v**_*ij*_*′* is the relative velocity of the *j*-th atom of molecule *i* relative to its center-of-mass velocity. The matrix _*i*_ is a 3 × 3 matrix containing the principal axes of inertia vectors of molecule *i* as its column vectors.

The projection operator Ξ_*ij*_ can then be derived as^32^
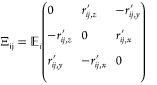
20with *r*_*ij*,*x*_*′*, *r*_*ij*,*y*_*′* and *r*_*ij*,*z*_*′* being again the positions of the *j*-th atom in molecule *i* relative to its center-of-mass.

A Ξ_*ij*_ operator is then generated for each atom *j* in molecule *i* along with its corresponding rotational velocity. With the latter quantities a total of 3*N*_atoms,*i*_ expressions like in formula 17 are generated for each molecule. Combining these expressions for all atoms *j* in molecule *i* the following matrix equation can be derived
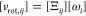
21where [*v*_rot,*ij*_] is a 3*N*_atoms,*i*_ × 1 matrix containing the rotational velocities and [Ξ_*ij*_] is a 3*N*_atoms,*i*_ × 3 matrix containing the projection operators of all atoms *j* in molecule *i*. [ω_*i*_] is a 3 × 1 matrix containing the angular velocity vector of molecule *i*. Now, in order to solve the equation for the angular velocities a square matrix of the projection operator matrix [Ξ_*ij*_] has to be generated in order to take the inverse of the matrix, which then results in the following expression
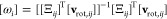
22With the resulting angular velocities the rotational kinetic energy of all molecules and the resulting effective rotational temperature of the system can be determined. The rotational scaling factor λ_rot_ can then be calculated and applied to the rotational velocity of each atom in the system.

In the last step, the vibrational velocities of the system can be determined by subtracting the translational and rotational components from the total velocity of each atom in the system. Then, with the available vibrational kinetic energy of the system and the resulting effective temperature the associated scaling factor λ_vib_ can be determined and applied to the vibrational velocity of each atom in the system.

The resulting scaled velocity of an atom in the simulation box is the sum of the scaled translational, rotational and vibrational components. Afterward, the scaled velocities of all atoms in the simulation box are used to perform the next time step of the MD simulation.
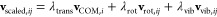
23

### 2.5 Intramolecular Virial Correction

In general, in MD simulations the pressure can be expressed via the virial theorem as given in eq 1. Therefore, the virial of the system can be considered as a measure for the internal pressure and can be expressed in general for orthorhombic systems as
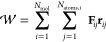
24with **r**_*ij*_ and **F**_*ij*_ being the position and force of the *j*-th particle in molecule *i*, respectively. Usually, the pressure in an MD simulation is treated on a molecular basis, thus, a certain intramolecular virial correction for each molecule has to be applied. While for pairwise additive force fields the potential energy can be directly separated into intra- and intermolecular contributions, this is generally not possible for QM-based simulations, hence a special treatment is required.

### 2.6 The Stress Tensor

To calculate the virial for QM-based simulations the stress tensor **σ** must be taken into account. For calculations in a periodic box the stress tensor is a direct output of the QM calculation. In general, for orthorhombic systems, the stress tensor has the following relationship considering the virial
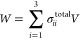
25where *W* is the total scalar virial of the system and *V* is the volume of the simulation box. σ_*ii*_^total^ represents the diagonal elements of the total stress tensor. In order to obtain a molecule-based virial description an intramolecular virial correction must be applied to each molecule. Therefore, for a total of *N* molecules in the simulation box further *N* single point calculations have to be performed per MD step, where each calculation considers only a single molecule of the system. The summation of the diagonal elements of the resulting stress tensors σ is then a direct measure for the intramolecular virial contribution. The intermolecular stress tensor σ^inter^ is then obtained by subtracting the intramolecular contributions from the total stress tensor of a QM calculation considering all molecules, i.e.,
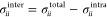
26The only restriction for this approach is that the dimensions of the periodic box must be sufficiently large to ensure interactions with their own periodic image are negligible.

This approach, which involves performing *N* further single point calculations on a QM-based description of the system in every MD step, is computationally highly demanding, even when semiempirical QM methods such as DFTB are used. The overhead of the additional *N* single point calculations is oftentimes not feasible for large systems, thus, a more efficient approach is needed. In order to reduce the computational effort, for small molecules the intramolecular virial correction can be approximated via an interpolation table.

### 2.7 Interpolation Table for Intramolecular Virial Contributions

For small molecules with few internal degrees of freedom an interpolation table can be constructed. Pure gaseous systems are usually calculated within cubic simulation cells, thus, the directional dependence of the virial and the stress tensor can be ignored. Therefore, the pressure of a cubic system can be calculated as follows
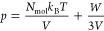
27This consideration enables the use of internal coordinates to build the interpolation table. Given a sufficiently large simulation cell, the orientation (i.e., the rotation) of the molecule does not change the overall intramolecular virial of the molecule. Considering a molecule with *d* internal degrees of freedom, a stress tensor grid of *d* dimensions can be constructed. A grid point represents a certain configuration of the molecule, uniquely described by its internal coordinates.

In the specific case of CO_2_, a single molecule has three internal degrees of freedom, namely the two C–O bonds and the O–C–O angle. This allows a three-dimensional lattice to be constructed. The interpolation table is then calculated by performing a single point calculation for each grid point, representing a certain displacement of the three internal coordinates, from its minimum energy configuration (via ). For the construction of an interpolation table, the resultant grid can be treated with an appropriate method, such as cubic spline interpolation. The latter can then be used to calculate the intramolecular virial correction of each molecule in its instantaneous configuration. The construction of this table needs to be carried out once per molecule species and applied level of theory, and can be performed independently prior to the execution of the MD simulation.

## 3 Simulation Setup

Generally, most of the simulations conducted in this work, were sampled using an NVT ensemble at 298.15 K. The temperature was controlled using the presented equipartitioning thermostat, unless otherwise stated. The force evaluation was performed using the dftb+ software package^33^ at DFTB3^34^/D3^35^ level of theory in conjunction with the 3ob parameter set.^26,36^ The simulations were performed using an in-house developed MD software package.^37−39^ The time step for all simulations was set to 2 fs, except for the evaluation of the methane isothermes, where a time step of 0.5 fs was used to account for the fast intramolecular vibrations. In Figure S1 in the Supporting Information, several molecular dynamics (MD) simulations are presented to assess whether a 2.0 fs time step is appropriate for the pristine CO_2_ gas by evaluating energy conservation under pressures of 1 and 10 bar. These results are compared with simulations using 0.1 and 1.0 fs time steps in a microcanonical (NVE) ensemble to ensure the 2.0 fs time step maintains accurate energy conservation.

## 4 Results and Discussion

### 4.1 The Equipartition Theorem

Although the equipartition theorem should be satisfied in thermal equilibrium without the necessity of a manual temperature steering of the different degrees of freedom, it can be shown, that this condition is not satisfied for gaseous systems treated at a QM level of theory such as DFTB3. This is demonstrated by performing five simulations of gaseous CO_2_ applying the velocity rescaling thermostat for temperature control.

A total of 50 CO_2_ molecules were placed into a cubic simulation cell of 100.9597 Å box length, with random velocity components according to the Maxwell–Boltzmann distribution^40^ corresponding to 298.15 K in the translational, rotational and vibrational degrees of freedom. The total linear momentum of all translational degrees of freedom was ensured to be zero. A pressure of about 2.0 bar is expected since these conditions are anticipated to cause CO_2_ to exhibit properties similar to those of an ideal gas. Furthermore, it has to be noted, that the first frame in all subplots of Figure 1 does not correspond to the initial frame but represents the physical observables obtained after the first MD step of 2.0 fs. Therefore, the initial temperatures of the five different runs do not correspond to the expected 298.15 K.

Figure 1a clearly demonstrates the time evolution of the instantaneous pressure of the system is not constant. In addition, all five simulations produce an average pressure within a range of 2.4 to 2.6 bar (+20–30%) that deviates significantly from the predicted ideal gas pressure of 2.0 bar, indicating that the simulations are far from being converged.

In order to investigate the origin of the observed pressure deviations, the time evolution of the total temperature of the system was considered. The latter was decomposed into its translational, rotational and vibrational contributions, shown in Figure 1b–d. As can be seen, the expected distribution of the temperature among the different degrees of freedom is not achieved. Especially, when comparing the rotational and vibrational temperature it can be noted, that the equipartition theorem is not satisfied. The rotational temperature is significantly higher than the vibrational temperature, which is not expected for a gaseous system at 298.15 K. Considering the small number of collisions between the gas molecules with respective large free mean path, the redistribution of the kinetic energy among the different degrees of freedom is comparatively slow. In case of the example shown in green it can be seen that probably due to a rare collision event at approximately 250 ps the equipartitioning between the vibrational and rotational temperature component is further de-equilibrated. The deviation in the different thermal components from the expected value of 298.15 K also has a negative impact on the translational temperature, which is directly responsible for the deviations observed in the pressure. To further analyze the de-equilibration phenomenon observed, kinetic energy decompositions were conducted using a Nosé–Hoover chain thermostat^41,42^ with a chain length of 5. These results are included in the Supporting Information in Figure S2. The observed behavior is consistent with that seen when using the stochastic canonical velocity rescaling thermostat, with similar patterns for time steps of 0.1 and 2.0 fs, as well as for pressures of 1 and 10 bar.

**Figure 2:**
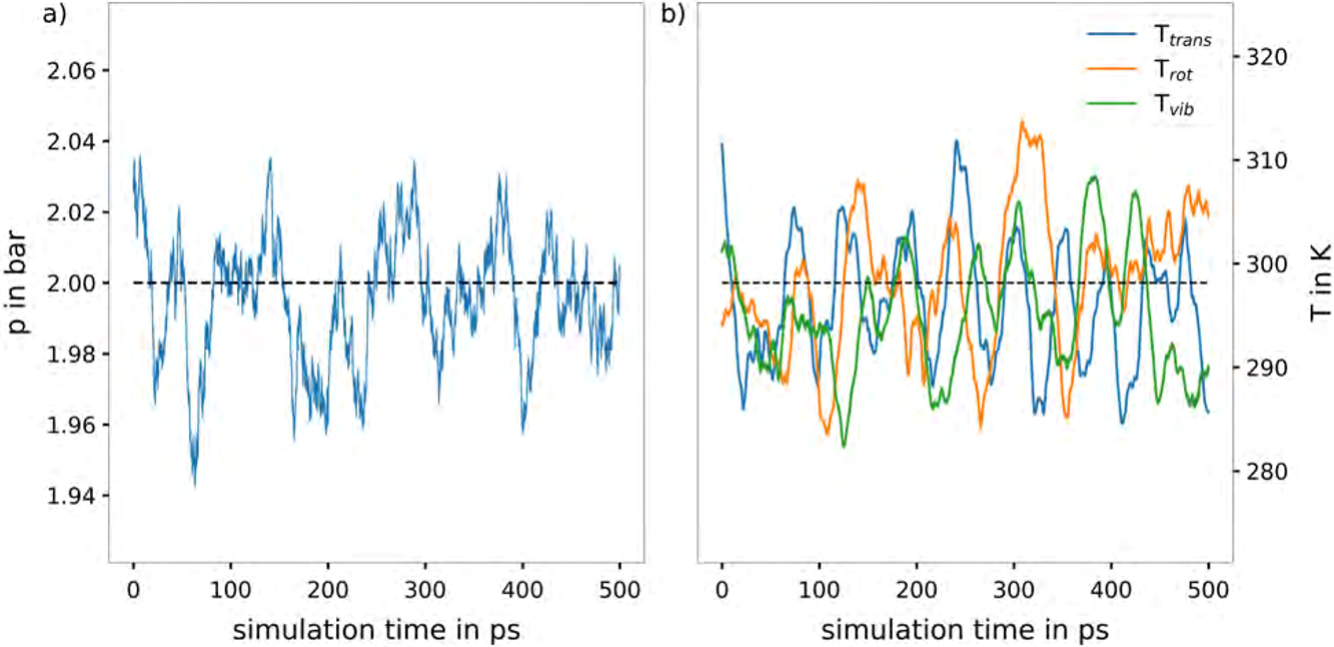
Time evolution of (a) pressure (blue) as well as (b) the translational (blue), rotational (orange), and vibrational (green) temperature obtained from an NVT QM-MD simulation at DFTB3/3ob/D3 level of theory of gaseous CO_2_ applying the newly developed equipartitioning thermostat to a cubic simulation cell with a box length of 100.9597 Å. The CO_2_ molecules were placed randomly in the simulation box and the velocities were initialized according to the Maxwell–Boltzmann distribution at 298.15 K. The expected ideal gas pressure of 2.0 bar and the target temperature of 298.15 K for all degrees of freedom are included as black dashed lines.

To ensure a correct distribution of kinetic energy among individual degrees of freedom within a feasible simulation time, the presented equipartitioning thermostat was developed. Literature indicates that violations of the equipartition theorem in classical force fields can be mitigated by using very large system sizes or exceptionally long simulations.^43^ However, these approaches are not feasible for QM-based methods, such as DFTB, due to their substantial computational demands. As a result, the proposed thermostatization strategy is designed to apply the stochastic canonical velocity rescaling thermostat independently to the translational, rotational, and vibrational degrees of freedom. The resulting time evolution of the instantaneous pressure of the system is shown in Figure 2a. As can be seen, the pressure of the system is quickly equilibrated and displays a much smaller magnitude in fluctuation. The average value in the second half of the simulation is in agreement with the predicted ideal gas pressure of 2.0 bar. The resulting time evolution of the total temperature of the system was considered as well. The latter was again decomposed into its translational, rotational and vibrational contributions, as shown in Figure 2b. Here, only a single simulation is shown, as the time evolution of the pressure and the different temperature components of the system is analogous for all 5 simulations. As can be seen, the expected distribution of the temperature among the different degrees of freedom is achieved in accordance with the equipartition theorem. The rotational and vibrational temperatures are in agreement with the translational temperature, which is expected for a gaseous system at 298.15 K. For a correct pressure treatment, all simulations were performed employing an intramolecular virial correction using the interpolation table scheme, which is discussed in the following.

### 4.2 Virial Interpolation Table

To address the problem of the intramolecular virial correction for QM-based simulations, an interpolation table was build for CO_2_. The latter was set up using a 3-dimensional grid, where each grid point represents a certain configuration of the molecule, uniquely described by its internal coordinates *r*_CO_1__, *r*_CO_2__ and α_OCO_. The resulting grid was interpolated via cubic spline interpolation, to calculate the intramolecular virial correction for each molecule in its instantaneous configuration.

As in NVT simulations the correction of intramolecular virial contributions does not affect the resulting configurations of the trajectory, but only the resulting pressure of the system, the previously introduced NVT simulations of randomly placed CO_2_ molecules in a cubic simulation box were considered to determine the distributions of the bond lengths and angle deformations of each CO_2_ molecule in the simulation box. The resulting distributions are shown in Figure S3 of the Supporting Information. Considering the minimal and maximal bond lengths of the distributions and the minimal angle in the O–C–O mode, an interpolation table with 10% margin was build in order to avoid any necessity of extrapolation when applying cubic splines to the interpolation grid.

**Figure 3:**
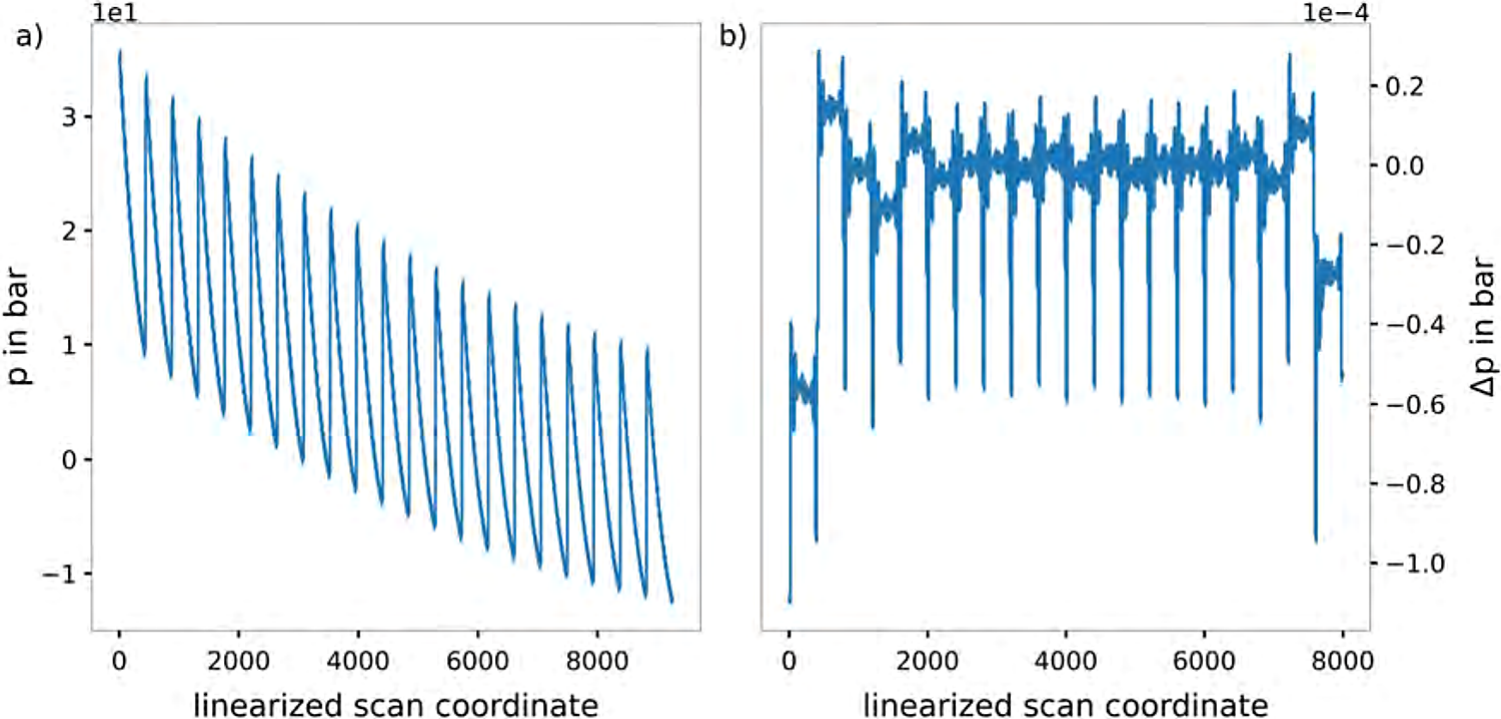
Comparison of the resulting pressure corrections of the benchmark configurations of the interpolation table and the explicit single point calculations using DFTB3/3ob/D3 level of theory. (a) depicts the absolute values of the pressure corrections of the benchmark configurations, while (b) shows the resulting pressure deviations obtained from the interpolation table compared to the explicit single point calculations projected onto a linearized scan coordinate.

In accordance with the determined ranges for the internal coordinates of a CO_2_ molecule, an interpolation table was built with a grid spacing of 0.1 Å for the bond lengths and 1° in case of angle deformation. Therefore, the resulting interpolation table was constructed by calculating the stress tensor for each molecular configuration using single point calculations at DFTB3/3ob/D3 level of theory with the same parameter set as used for the MD simulations in a simulation box with box length of 100 Å. In order to verify the accuracy of the interpolation table, a new set of configurations was generated exactly in the center of each cube of the interpolation grid, where in general the largest deviations are expected. In other words, the generated CO_2_ configurations displayed bond lengths and angles, that were fixed to the center of their respective values used for the construction of the interpolation table. Then, for each of these configurations the resulting intramolecular virial and its corresponding pressure were calculated using the interpolation table and compared to the resulting pressure of explicit single point calculations (i.e., one virial calculation per molecule and MD step). The resulting pressure corrections of the benchmark configurations are shown in Figure 3a, while the resulting deviations are shown in Figure 3b. Theoretically, the comparison of the resulting pressure and pressure deviations would need a four dimensional representation, however, for the sake of simplicity the results are shown in a two-dimensional plot with a linearized *x*-axis depicting the three internal coordinates of the CO_2_ molecule. From Figure 3b it can be stated that the interpolation table is in good agreement with the explicit single point calculations, as the resulting pressure deviations do not exceed an absolute value of 1.2 × 10^–4^ bar.

Finally, the volume (i.e., cell size) dependence of the intramolecular virial correction was considered, in order to be generally applicable to different simulation cells. To derive a quantitative description of the latter dependence a total of 20 different cubic simulation cells with lattice parameters ranging from 20 to 200 Å were constructed, containing a single CO_2_ molecule featuring always the same configuration. Afterward, the intramolecular virial was calculated for each of these simulation cells using explicit DFTB3/3ob/D3 single point calculations. Figure S4 of the Supporting Information shows the resulting inverse volume dependence of the absolute pressure corrections derived from the intramolecular virial contributions. As already pointed out in the Section 2, all included simulation box sizes were chosen to be sufficiently large to ensure interactions with their own periodic image are negligible. A linear fit of the resulting data points was performed, resulting in a perfect linear correlation with an *R*^2^ value of 1.00. Therefore, only a single interpolation table has to be constructed for a certain molecule species and applied level of theory, independently of the simulation box size, thus not only enabling the different NVT ensemble simulations, but also giving direct access to the intramolecular virial correction during NPT simulations, where the volume of the simulation box is allowed to fluctuate.

### 4.3 Isothermes

**Figure 4:**
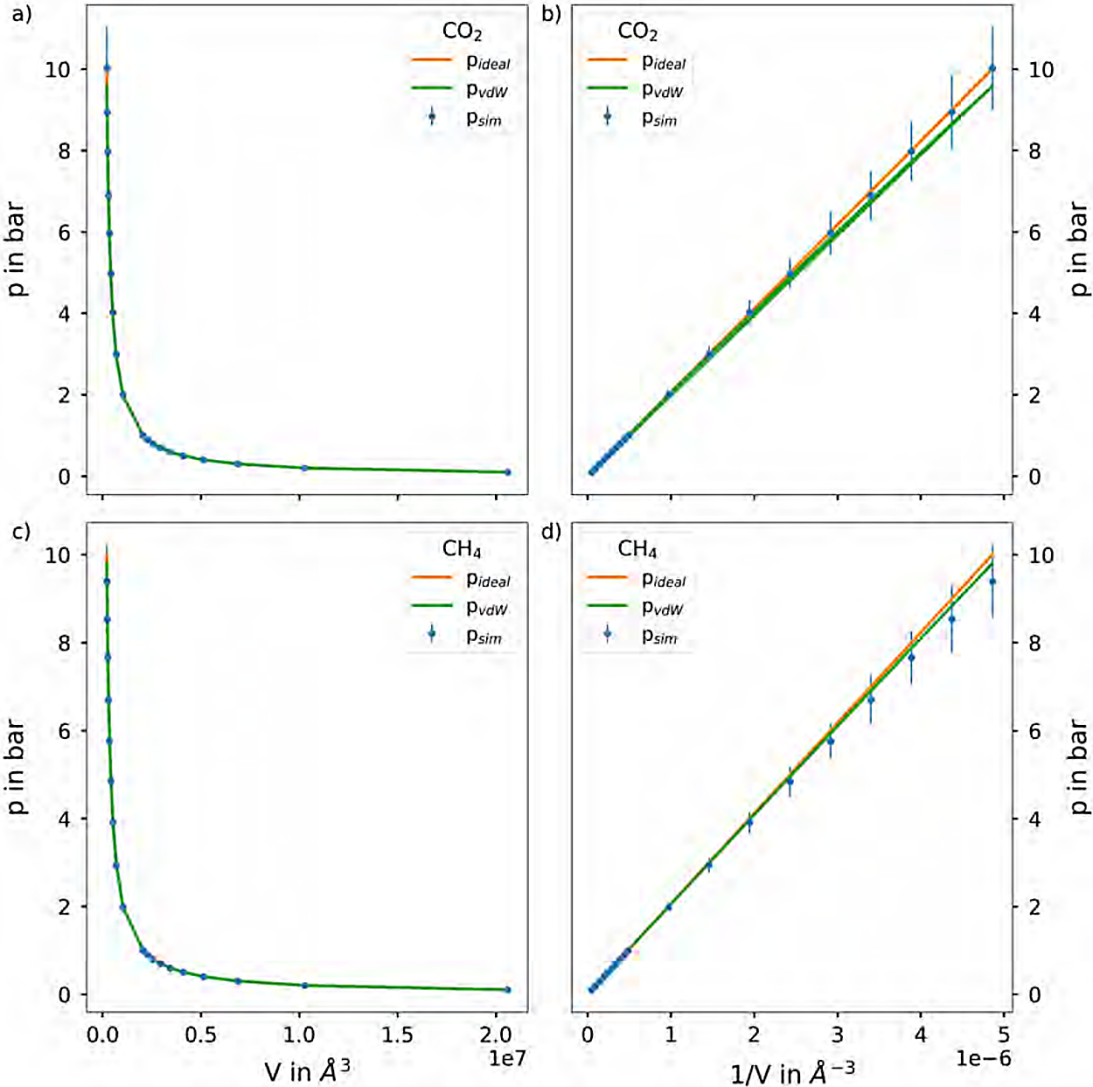
pV-diagrams of gaseuous (a) CO_2_ and (c) CH_4_, along with the expected behavior of an ideal and a van der Waals gas. The error bars are calculated via the standard deviation of the average pressures. (b and d) show the linearized version of the pV-diagrams of gaseous CO_2_ and CH_4_, respectively, along with the linearized version of the expected behavior of an ideal and a van der Waals gas. Each of the state points of gaseous CO_2_ and CH_4_ were determined at 298.15 K in an NVT ensemble applying the equipartitioning thermostat to cubic simulation cells with box lengths determined by the ideal gas law.

In order to test the applicability of the presented equipartitioning thermostat, the isothermes of gaseous CO_2_ and CH_4_ were considered. Therefore, 19 NVT QM-MD simulations at DFTB3/3ob/D3 level of theory were conducted for both gaseous CO_2_ and CH_4_ applying the equipartitioning thermostat for a temperature of 298.15 K. Each simulation was performed in cubic simulations boxes where the box length was determined by the ideal gas law for pressures ranging from 0.1 to 1.0 bar with a step size of 0.1 bar, for pressures ranging from 2.0 to 10.0 bar with a step size of 1.0 bar and a fixed number of molecules, specifically 50 CO_2_ and CH_4_ molecules, respectively. All simulations were performed for 300 ps of equilibration with additional 500 ps of sampling time. Each of the resulting average pressures from the sampling phase can then be mapped to one state point in the pV-diagram. The resulting pV-diagrams of gaseous CO_2_ and CH_4_ are shown in Figure 4a,c, respectively. In addition, pV-diagrams of the expected behavior of an ideal and a van der Waals gas^44^ are included. Furthermore, also the error bars calculated via the standard deviation of the average pressures are shown for each simulation. The van der Waals equation for the pressure of a gas is given by
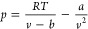
28where *R* is the universal gas constant, *T* is the temperature and *v* is the molar volume. The van der Waals constants for CO_2_ and CH_4_ are *a* = 3.640 L^2^ bar mol^–2^, *b* = 0.04267 L mol^–1^ and *a* = 2.253 L^2^ bar mol^–2^, *b* = 0.04278 L mol^–1^, respectively.^45^ The resulting linearized version of the pV-diagram of gaseous CO_2_ and CH_4_ are shown in Figure 4b,d, respectively, along with the expected linearized behavior of an ideal and a van der Waals gas. As can be seen, the resulting pV-diagrams of gaseous CO_2_ and CH_4_ are in good agreement with both the expected behavior of an ideal and a van der Waals gas, especially in the low pressure range.

Comparing the behavior of the linearized version of the *pV*-diagrams of gaseous CO_2_ with the expected behavior of a van der Waals gas it can be stated that the resulting pressures from the MD simulations are in general higher than the predicted van der Waals behavior. From this deviation it can be concluded that DFTB3/3ob/D3 level of theory underestimates the intermolecular interactions of gaseous CO_2_ resulting in more ideal gas like behavior. For gaseous CH_4_ the resulting pressures from the MD simulations are in general lower than the predicted van der Waals behavior. From this deviation it can be concluded that the applied level of theory contrarily to the case of gaseous CO_2_ overestimates the intermolecular interactions of gaseous CH_4_ resulting in less ideal gas like behavior. Nevertheless, the observed behavior in the low pressure range clearly indicates that the equipartition thermostat is capable of providing adequate estimates for the pressure in the simulation. On the other hand, the van der Waals law only provides an approximate equation of state and is known to also show deviations upon increasing pressures.

### 4.4 Gaseous CO_2_ and CO_2_@MOF-5

**Figure 5:**
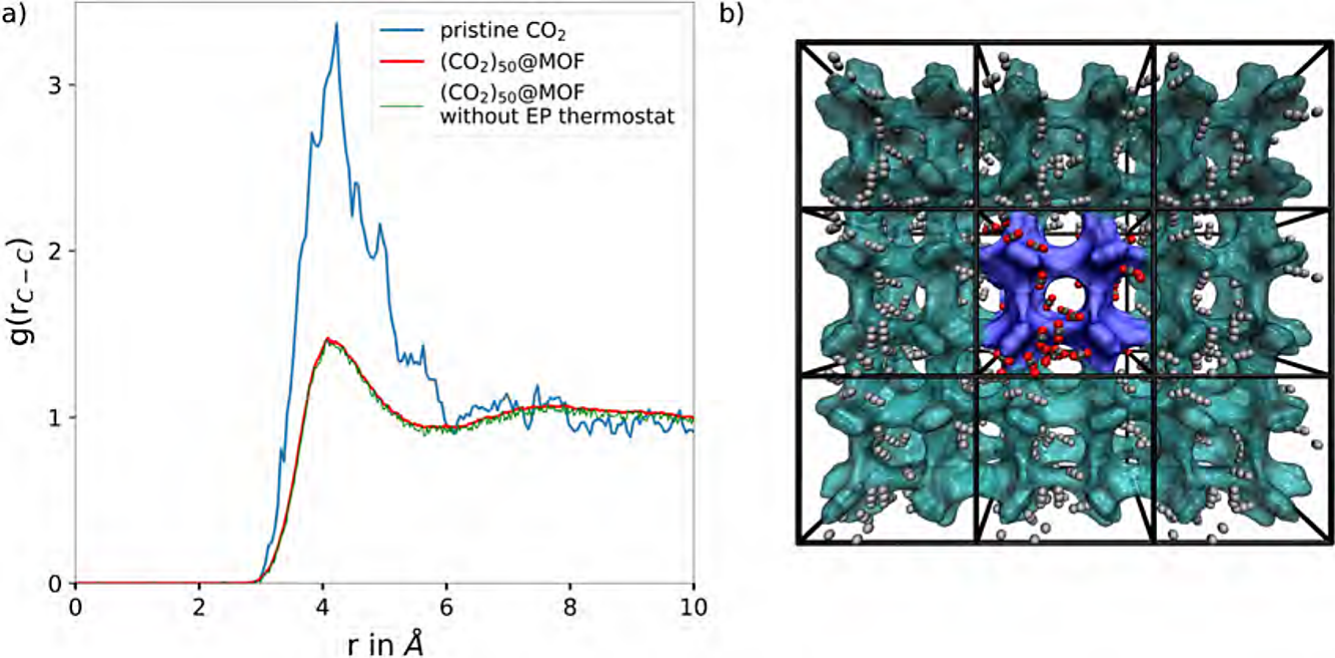
(a) Comparison of the C_CO_2__–C_CO_2__ radial distribution functions of pristine gaseous CO_2_ (blue) and (CO_2_)_50_@MOF-5 host–guest system (red/green). For both systems the RDFs were determined from MD-simulations at DFTB3/3ob/D3 level of theory in conjunction with the Berendsen weak coupling manostat.^31^ For the pristine CO_2_ only the presented equipartitioning thermostatization strategy and for the MOF-5 host–guest system both the stochastic canonical velocity rescaling thermostat^29^ and the newly developed equipartitioning thermostat were applied. The RDFs were determined from 500 ps sampling phase of the simulations at 298.15 K and 1.0 bar. (b) One sampled configuration of (CO_2_)_50_@MOF-5 host–guest system, where the dark blue highlighted center cube represents the simulation cell containing 50 CO_2_ molecules in a MOF-5 framework. To illustrate the periodic nature of the porous host framework, additional periodic images in *x*- and *y*-direction are included.

One further proof of concept is the analysis of radial distribution functions (RDFs) of pristine gaseous CO_2_ compared to CO_2_ molecules embedded in the metal–organic framework MOF-5.^13,46−49^ These RDF analyses are a direct measurement of the spatial distribution of the gas molecules in the simulation system. For the determination of the RDFs the 500 ps sampling phase of the simulation at 298.15 K and 1.0 bar, included as a state point in the isothermes shown in Figure 4a,b, was considered. For the (CO_2_)_50_@MOF-5 host–guest system two different NPT simulations were performed both resulting in 500 ps of simulation time using the Berendsen manostat.^31^ One of the latter two simulations was performed using the stochastic canonical velocity rescaling thermostat,^29^ while the second simulation was performed applying the presented newly developed equipartitioning strategy. MOF-5 represents a typical framework for gas storage applications, were the CO_2_ molecules show a characteristic being similar to the liquid state, i.e., the formation of a second solvation shell peak near approximately 7.6 Å^46^ and therefore featuring a different RDF compared to the gaseous state. From Figure 5a, where all three simulations, i.e. the gaseous system as well as both (CO_2_)_50_@MOF-5 simulations are shown, it can bee seen, that the gaseous system features a first shell at 4.1 Å, similar to the first shell of the liquid-like state. However, the gaseous state shows no second shell at around 7.6 Å, which is a clear indication of the different spatial distribution of the liquid nature of the MOF-5 framework. Furthermore, the radial distribution functions of the CO_2_ molecules in the MOF-5 framework show no significant difference between the stochastic canonical velocity rescaling thermostat and the newly developed equipartitioning thermostatization strategy, which corresponds exactly to the predicted behavior as in the liquid like state the redistribution of the kinetic energy among the different degrees of freedom is comparably fast, due to the high number of collisions between the liquid-like gas molecules, as well as with the host atoms of the MOF-5 framework.

The presented simulations have shown that the radial distribution function of carbon–carbon atoms in CO_2_ guest molecules remains consistent, regardless of the specific thermostatization strategy employed. This consistency is particularly important when utilizing a hybrid computational approach that combines classical MD with Grand Canonical Monte Carlo (GCMC) simulations. In this framework, a pristine CO_2_ gas system serves as a reference point, which is crucial for determining properties such as absorption isotherms in host systems like MOFs. The primary advantage of this hybrid method is its ability to allow the host material to adapt its structure in response to the number of guest molecules present. This adaptability is typically not accounted for in traditional GCMC simulations, where the host structure is often considered rigid. Therefore, analyzing the behavior of MOF systems with guest molecules is vital to demonstrating the broad applicability of the presented methodology across different phases and conditions, extending beyond the specific case of pristine gas systems. Although the Berendsen manostat is known to not yield the exact NPT ensemble, it was selected to replicate the simulation settings described in a previous work.^46^ Moreover, the resulting RDFs primarily serve as a proof of concept for the newly introduced thermostatization strategy as described above. In addition, solid-state systems typically display less pronounced fluctuation in the cell parameters in NPT simulations, thus, reproducing the exact ensemble was not a priority in this case.

## 5 Conclusions

In this work, a new thermostatization approach based on the equipartition theorem is presented. In thermal equilibrium, the equipartition theorem should be satisfied without the need for manual temperature control of the different motions. However, it has been discovered that for gaseous systems with a low number of collisions, resulting in a large free mean path, the equipartition theorem cannot be satisfied within a feasible simulation time when using (semiempirical) QM-based MD simulations (in this study, DFTB3) of gaseous systems. The presented equipartitioning thermostat is based on the stochastic canonical velocity rescaling thermostat by Bussi and co-workers,^29^ where the latter strategy is applied to the translational, rotational and vibrational temperature separately. The equipartitioning thermostat correctly distributes the kinetic energy among different degrees of freedom within a reasonable simulation time, as demonstrated by the comparison with simulations using the stochastic canonical velocity rescaling strategy. The resulting pV-diagrams of gaseous CO_2_ and CH_4_ exhibit the expected behavior of ideal and van der Waals gases. The radial distribution functions of CO_2_ molecules were compared between their gaseous state and their state embedded in a MOF-5 framework. The gaseous state exhibits a distinct spatial distribution in comparison to the liquid-like state observed within the MOF-5 framework. The radial distribution functions of CO_2_ molecules in the MOF-5 framework show no significant difference between the stochastic canonical velocity rescaling thermostat and the newly developed equipartitioning strategy. This result aligns with the predicted behavior. The equipartitioning thermostat presented here is an essential tool for simulating gaseous systems at quantum mechanical level of theory. This is especially relevant for simulations involving gaseous systems with a low number of collisions, such as those encountered in gas storage applications of MOFs, treated with Grand Canonical Molecular Dynamics (GCMD)^50^ simulations, where an external gas reservoir is considered. Furthermore, the treatment of the pressure calculation via the stress tensor and therefore via the virial theorem is a valuable tool considering most of the QM-MM-based MD implementations, where oftentimes it is not evitable to combine the MM and QM treatment of the systems via an adequate description of the virial.

Even though the equipartition thermostat was presented with a focus on DFTB (QM-based) simulations, where longer simulation times are often impractical due to substantial computational demands, it also offers significant benefits for classical simulations. By reducing equilibration times from several tens of nanoseconds to just a few picoseconds, it markedly enhances the efficiency of classical MD simulations. This makes the presented thermostatization strategy a valuable tool not only for QM-based simulations, where extended equilibration is challenging, but also for classical molecular mechanics (MM) simulations, where it can greatly streamline the equilibration process.

## Data Availability

All calculations have been conducted using the PQ^39^ and PQAnalysis^51^ software packages, which are available at https://github.com/MolarVerse/PQ and https://github.com/MolarVerse/PQAnalysis, respectively. At present the outlined equipartitioning thermostat is implemented in a development branch of the PQ software package and will be made available in the near future.

## Supporting Information Available

NVE energy conservation regarding time step and pressure dependency; translational, vibrational and rotational temperature time evolution using the Nosé–Hoover chain thermostat; representation of C–O bond length and O–C–O angle distributions of CO_2_ for interpolation table construction; volume and inverse volume dependency of pressure corrections for interpolation table construction (PDF)
