# Supplementary material for: Equipartitioning of Molecular Degrees of Freedom in MD Simulations of Gaseous Systems via an Advanced Thermostatization Strategy
Source: J Chem Theory Comput. 2024 Dec 19;21(1):102–13. doi: 10.1021/acs.jctc.4c01580 (PMC11736790; doi:10.1021/acs.jctc.4c01580)
Supplement: Supplementary file 1 — ct4c01580_si_001.pdf [file ct4c01580_si_001.pdf]

Equipartitioning of Molecular Degrees of Freedom in MD Simulations of Gaseous Systems *via* an Advanced Thermostatization Strategy

Jakob Gamper,[†] Josef M. Gallmetzer,[†] Risnita Vicky Listyarini,[†] Alexander K. H. Weiss,[‡] and Thomas S. Hofer[*,†]

†*University of Innsbruck, Theoretical Chemistry Division, Institute of General, Inorganic and Theoretical Chemistry, Center for Chemistry and Biomedicine, Innrain 80-82, A-6020 Innsbruck, Austria*

‡*University of Innsbruck, Research Institute for Biomedical Aging Research, Rennweg 10, A-6020 Innsbruck, Austria*

E-mail: t.hofer@uibk.ac.at

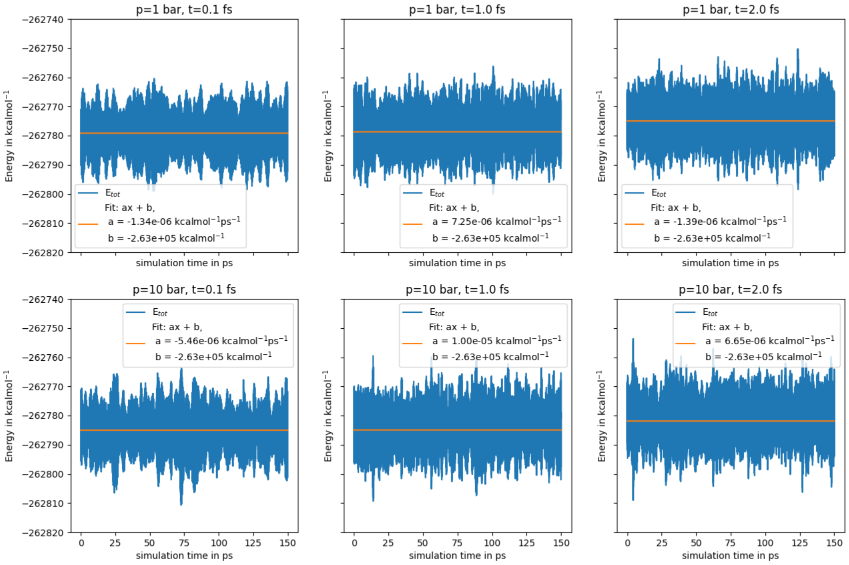

Figure S.1: Time evolution of the total energy (blue) from six different NVE QM-MD simulations of gaseous CO2 at DFTB3/3ob/D3 level of theory for box lengths corresponding to 1 and 10 bar at 298.15 K and time steps of 0.1, 1.0 and 2.0 fs. In orange a linear fit of the total energy is shown in order to visualize the energy drift over the simulation time.

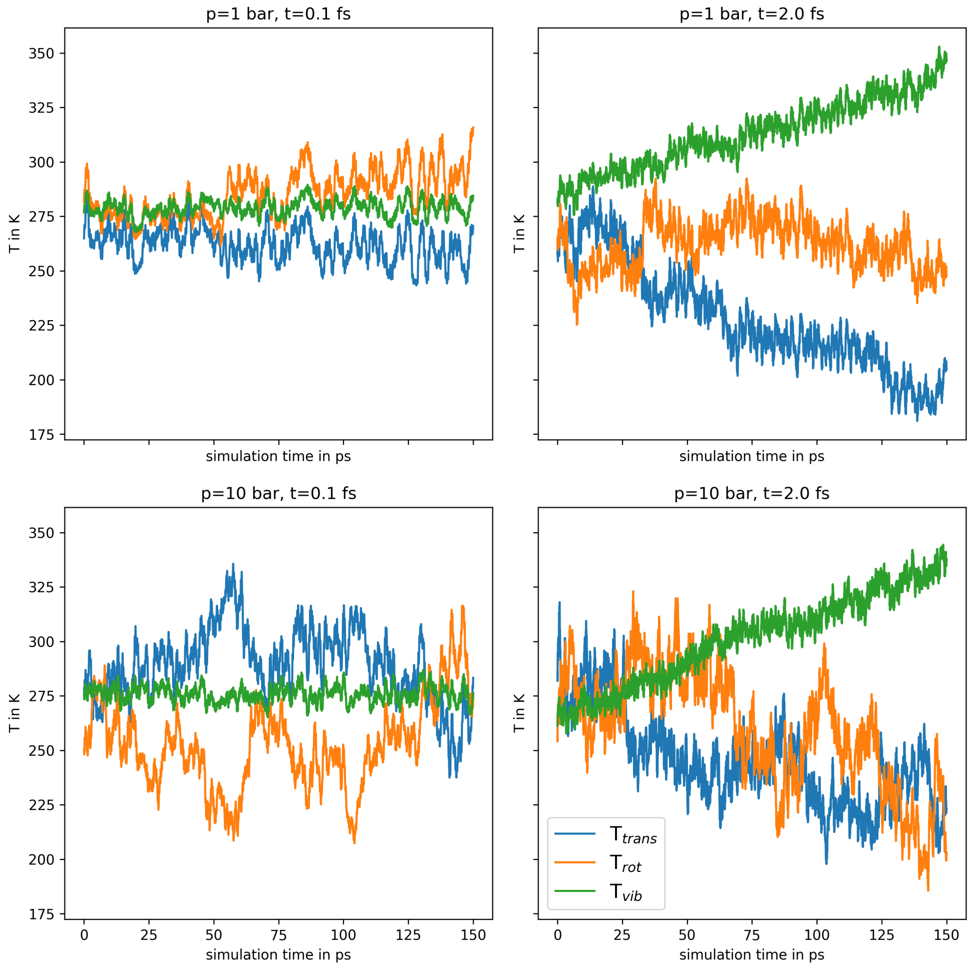

Figure S.2: Time evolution of the translational, rotational and vibrational temperatures obtained from four different NVT QM-MD simulations of gaseous CO2 at DFTB3/3ob/D3 level of theory applying the Nose-Hoover thermostat to a cubic simulation box with box lengths corresponding to pressures of 1 and 10 bar at 298.15 K and time steps of 0.1 and 2.0 fs.

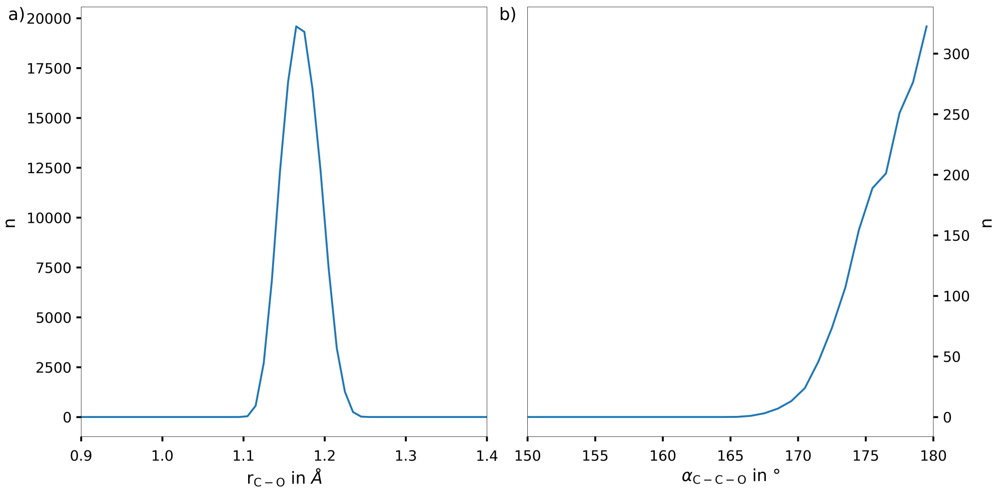

Figure S.3: Distribution of a) C−O bond lengths and b) O−C−O bond angles of gaseous CO_2 containing 50 molecules from 500 ps of sampling time at 298.15 K applying the presented equipartition thermostatization strategy. The simulation was conducted at DFTB3/3ob/D3 level of theory using an NVT ensemble with a time step of 2.0 fs.

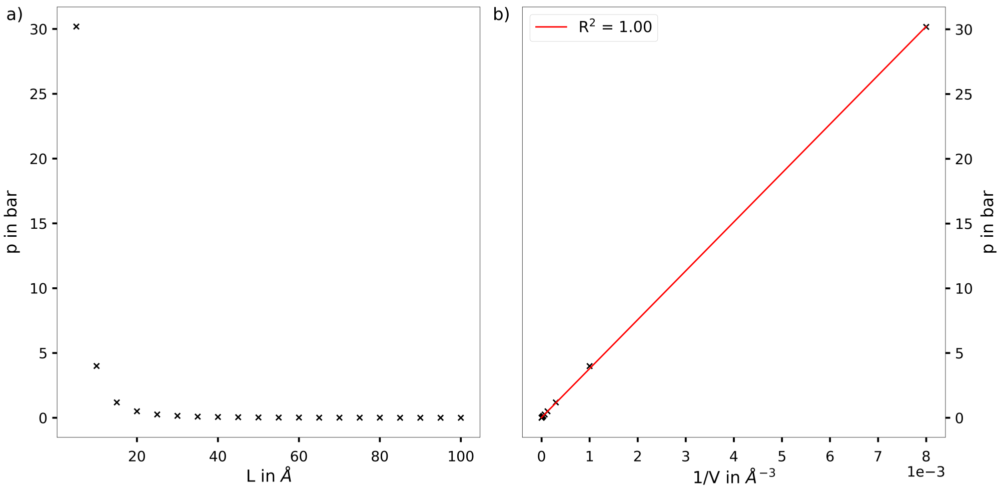

Figure S.4: Simulation cell size dependency for a total of 20 different lattice parameters of the absolute pressure value for a single CO_2 molecule using single point calculations at DFTB3/3ob/D3 level of theory. a) depicts the pressure dependency of the applied simulation box lengths, while b) shows the pressure dependency of the inverse volume of the simulation cell including a linear fit resulting in a perfect linear correlation with R^2 of 1.00.
